# Hypothyroidism in Patients with Down Syndrome: Prevalence and Association with Congenital Heart Defects

**DOI:** 10.3390/children11050513

**Published:** 2024-04-25

**Authors:** Francesca Gorini, Alessio Coi, Anna Pierini, Nadia Assanta, Antonio Bottoni, Michele Santoro

**Affiliations:** 1 Unit of Epidemiology of Rare Diseases and Congenital Anomalies, Institute of Clinical Physiology, National Research Council, 56124 Pisa, Italy; 2 Foundation Gabriele Monasterio CNR-Regione Toscana, 56124 Pisa, Italy; 3 Foundation Gabriele Monasterio CNR-Regione Toscana, 54100 Massa, Italy

**Keywords:** Down syndrome, congenital hypothyroidism, overt hypothyroidism, congenital heart defects, population-based study

## Abstract

This population-based study aimed to assess the prevalence of congenital hypothyroidism (CH) and overt hypothyroidism (OH) and their association with congenital heart defects (CHDs) in patients with Down syndrome (DS). The population included all live births residing in Tuscany (Italy) diagnosed with DS recorded in the Registry of Congenital Defects and in the Registry of Rare Diseases of Tuscany in the years 2003–2017. The prevalence of CH and OH in DS patients was calculated by sex and by period. The association of CH and OH with CHDs in DS patients was assessed using multivariate logistic regression. The cohort included 228 subjects. The prevalence of CH and OH was 11.4% (95%CI: 7.4–16.7%) and 12.7% (95%CI: 8.5–12.3%), respectively, with no significant difference by sex. A significant increase in the prevalence of CH (*p* < 0.0001) was found in the years 2010–2017 compared to the previous period, and among preterm infants (*p* = 0.009). The presence of CH was associated with a higher prevalence of CHDs (adjusted OR = 2.24, *p* = 0.082). A significant association between ventricular septal defects (VSDs) and the occurrence of OH (adjusted OR = 3.07, *p* = 0.025) was also observed. This study confirmed the higher prevalence of both CH and OH in DS compared to the general population. Furthermore, the risk of association between DS and CHDs was higher in the presence of CH, while VSDs are associated with OH, providing relevant insights into the epidemiology of hypothyroidism in DS and associated anomalies.

## 1. Introduction

Down syndrome (DS) is the most frequent genetic disorder determined by abnormalities in the number or structure of chromosome 21 [1], with an estimated prevalence of 9.98 per 10,000 live births in Europe [2]. 

DS is associated with cognitive impairments and multiple medical conditions including congenital anomalies and thyroid disorders [3,4]. Among the concurrent medical diagnoses associated with DS, congenital heart defects (CHDs) are notoriously the leading cause of mortality and morbidity in patients with DS, with an estimated prevalence of 43.6% [3,5]. 

Patients with DS are also at risk of developing thyroid dysfunction that in DS can be autoimmune- or non-autoimmune-related, embracing a wide spectrum of disorders including congenital hypothyroidism (CH), overt hypothyroidism (OH), subclinical hypothyroidism (SH), autoimmune thyroiditis and hyperthyroidism [4,6]. 

Characterized by elevated plasma levels of thyroid-stimulating hormone (TSH) and low or normal thyroxine (T4) concentration at birth, CH is generally diagnosed shortly after birth through neonatal metabolic screening or within 6 months of life [7,8]. Since thyroid hormones play a crucial role in brain development in fetal and neonatal life, CH is considered one of the possible factors contributing to intellectual disability in DS patients and may influence their somatic growth and psychomotor development [9,10]. However, access to timely diagnosis and timely treatment with levothyroxine (L-T4), the exogenous form of T4, prevent these adverse effects and lead to normal neurologic growth [11]. 

The incidence of CH has increased in recent decades globally, including in Italy, with rates ranging from 1:3000–1:4000 to 1:1090–1:2800 due to lowering of cutoffs for TSH screening, changes in the ethnic composition of the population, the increased number and survival of preterm infants at high risk of CH, changes in clinical practice, and iodine deficiency [12,13,14,15]. Since iodine is an essential constituent of thyroid hormones, iodine deficiency is a relevant risk factor for CH, especially in iodine-deficient areas [11]. Of note, CH is classified into the two categories of transient and permanent CH, based on the need for temporary or lifelong treatment of the disease to restore normal thyroid function [16]. 

OH, which is characterized by low T4 levels and TSH levels greater than 10.1 mU/L, has an estimated prevalence of 0.3–5.3% in the general population [17], with development at any time of life [7,18]. In iodine-rich areas, the most common cause of OH is autoimmune thyroiditis, i.e., Hashimoto’s disease, which has as its major features the diffused infiltration of lymphocytes into the gland and the presence of thyroid autoantibodies [19,20]. As with CH, thyroid hormone replacement therapy with L-T4 is considered as the gold standard therapy for the treatment of OH [17]. A close association between CH and extrathyroidal anomalies, including CHDs, has been widely recognized, suggesting the possible role of common genes or intrauterine factors in multiple organ differentiation [21,22,23,24,25]. 

The aim of this population-based study was to estimate the prevalence of CH and OH in children with DS in Tuscany (Italy), also exploring their association with CHDs. 

## 2. Materials and Methods

### 2.1. Study Patients and Data Collection

In this retrospective study, the population included all liveborn cases with DS diagnosed in the years 2003–2017 and residing in Tuscany, an Italian region of 3,701,343 inhabitants (source: Italian National Institute of Statistics as of 1 January 2018). DS cases were extracted from the Registry of Congenital Defects of Tuscany (RCDT) and the Registry of Rare Diseases of Tuscany (RRDT). In both registries, cases are provided with a pseudonymized code. Cases with the same code were included only once in our study. RCDT is a population-based registry that collects cases of congenital anomalies among live births, fetal deaths after 20 weeks of gestational age and terminations of pregnancy after a prenatal diagnosis of fetal anomaly, diagnosed by a widespread network within the first year of life. This registry is a full member of the European network of population-based registries for the epidemiological surveillance of congenital anomalies (EUROCAT) [26] since 1979 and of the International Clearinghouse for Birth Defects Surveillance and Research since 1998. RRDT is a population-based registry active since 2005 and based on a regional network of health centers, some of which are centers of expertise. Six DS children who died within the first year of life were excluded from the study. 

In accordance with the classification used by EUROCAT [27], information on CHD co-occurrence in DS children was recovered by selecting Q20-Q26 codes (International Classification of Diseases—version 10—British Pediatric Association extension—ICD10-BPA) in RCDT. 

Cases with CH were identified, exploiting two concomitant criteria: (1) presenting E030 or E031 code in RCDT or having a diagnosis for CH in RRDT or having a co-payment exemption for CH; (2) having received at least one prescription of L-T4 (Anatomical Therapeutic Chemical—ATC code: H03AA01) in the study period. Children with transient CH were considered as cases who discontinued L-T4 therapy after the age of 3 years, according to criteria published by the European Society for Pediatric Endocrinology and the American Academy of Pediatric guidelines [28,29], and were excluded from the study (n = 8).

Cases with OH were instead identified as cases who had received at least one prescription of L-T4 per year in the study period without a diagnosis of CH, as defined before.

Furthermore, a random sample of 30,379 singleton liveborn infants without congenital anomalies, corresponding to 10% of all live births born in Tuscany in the years 2003–2017, was selected as the reference population. 

The following maternal covariates recovered from RCTD were considered: maternal age, gestational age, maternal body mass index (BMI), maternal education, maternal nationality and smoking during pregnancy. Maternal and paternal age were stratified into three levels: <35 years, ≥35–<40 years and ≥40 years. Gestational age was divided into two categories: ≥37 weeks (full term infants) and <37 weeks (preterm infants). Four levels were defined for BMI: <19 (underweight), ≥19–<25 (normal weight), ≥25–≤30 (overweight) and >30 (obese). Maternal education was divided into three categories: low (primary and lower secondary level), medium (upper secondary level) and high (tertiary level). As for maternal nationality, two groups were considered: Western Countries, including Italy, and other Western Developed Countries and High Migratory Outflow Countries that, in Tuscany, are mainly represented by Morocco, Albania and China. The variable tobacco smoking habits during pregnancy were stratified into nonsmokers and smokers (average daily cigarette consumption ≥ 1 cigarette). 

### 2.2. Statistical Analysis

Continuous variables were presented as means ± standard deviations (SDs), while categorical variables were expressed as absolute value and percentage. Proportions were used to estimate the prevalence of CHDs, CH and OH in total DS patients, by sex and by period (years 2003–2009; 2010–2017). Categorical variables were compared using the Chi-square test or Fisher’s Exact test. 

The association of CH and OH with CHDs in DS patients was estimated using multivariate logistic regression excluding OH and CH cases, respectively, from the two analyses. Crude and adjusted Odds Ratios (ORs) with a 95% confidence interval (95%CI) were calculated and a two-sided *p*-value < 0.05 was considered statistically significant. Data were analyzed using Stata, version 16 [30].

## 3. Results

The cohort included 228 DS subjects, of which 50.4% were males. CH and OH were diagnosed in 26 (prevalence 11.4%, 95%CI 7.4–16.7%) and 29 children (prevalence 12.7%, 95%CI 8.5–18.3%), respectively. The estimated prevalence of CH among newborns without congenital anomalies was 7 per 10,000 (95%CI 4–10). 

In our cohort, we also observed a substantial increase (*p* < 0.0001) in CH prevalence in 2010–2017 compared to the previous study period (15.0% vs. 7.4%). The increase in the more recent period was also confirmed in both sexes (Table 1). Conversely, for OH, we found no significant difference by period (Table 2).

The diagnosis of CH in our cohort occurred, on average, within 6–7 months of life, while the age of diagnosis of OH among DS children was highly variable, ranging from 1 month to 12 years (data not shown). 

No significant difference by sex was found in either CH or OH children. A higher proportion of premature infants (42.3% vs. 18.8%, *p* = 0.009) was recorded among children with CH compared to those without CH. No significant difference in the prevalence for either CH or OH was observed across maternal and paternal age, maternal nationality, BMI and smoking during pregnancy. Additionally, a significantly higher prevalence of CHDs was detected among DS patients with CH compared to subjects without CH (57.7% vs. 31.5%, *p* = 0.009) (Table 3). 

A total of 84 DS patients, corresponding to a prevalence of 36.8% (95%CI 29.4–45.7%), received a diagnosis for CHDs. A severe cardiac defect was found in approximately one in three subjects (32.1%). The most common CHD subgroups in DS patients were ventricular septal defects (VSDs) (16.2%), atrial septal defects (ASDs) (8.3%) and, among severe CHDs, atrioventricular septal defects (8.7%) (Table 4). 

Among children with CHDs, 17.9% and 16.7%, respectively, also had a diagnosis of CH and OH (57.7% and 48.2% of the total cases of CH and OH, respectively). In addition, 19.2% of DS patients with CH and 10.3% of those with OH had a severe CHD. The presence of CH significantly increased the prevalence of CHDs (OR = 2.96, 95%CI: 1.27–6.89, *p* = 0.012) and an association was also observed in the adjusted model (adjOR = 2.24, 95%CI: 0.90–5.58, *p* = 0.082). A significant association was found between OH and VSD (adjOR = 3.07, 95%CI 1.15–8.15, *p* = 0.025). No other associations can be observed with any of the CHD subgroups (Table 4).

## 4. Discussion

The increased prevalence of hypothyroidism—both congenital and acquired disorders—in patients with DS has been largely documented. In this population-based study, we estimated the prevalence of CH and OH at 11.4% and 12.7%, respectively. These prevalence estimates are difficult to compare to those of previous investigations using a hospital-based approach. Looking at the literature, the reported prevalence of CH in DS ranges from 2% to 16.5%, the prevalence of OH between 1% and 32.9% [6,7,8,31,32,33,34,35], and this high variability might depend on diagnostic tests and criteria for diagnosis, sample size, and time frames when the studies were conducted [29]. 

Our estimate of CH prevalence in the general population during 2003–2017 (0.07 cases per 1000 births), although higher than the most recent estimate reported in Italy (1:2000–1:3000 neonates), is half of the one registered in another Italian region, even if relating only to the years 2013–2018 [36]. In our cohort of DS patients, the number of CH cases detected in the years 2010–2017 doubled compared to the previous period, in line with the significant increase in CH incidence recorded in the general population of several countries [12]. Indeed, in recent years, due to changes in screening strategies and the introduction of progressively lower cutoffs of TSH to improve sensitivity for CH (i.e., from above 20–25 mIU/L to above 6–10 mIU/L), a greater number of mild forms of CH (some of which are transient) have been reported to be associated with a eutopic thyroid gland [37,38,39]. Nonetheless, a better tracking of CH cases cannot be ruled out, which also might explain the slight decrease in the prevalence of OH in more recent years. On the other hand, clinical signs of DS may interfere with a diagnosis of mild CH, thus leading to an underestimation of CH in these patients [25,40]. 

Of note, the increased prevalence of CH observed in our cohort in the last period may also be related to a growing incidence of preterm births and survival of very-low-birth-weight infants of around 20% over the last 20 years [41]. In this study, we observed a higher proportion (42.3%) of preterm infants among children with CH than in those without CH (18.8%). Preterm babies, and especially the smallest and less mature ones, have a higher risk of CH due to insufficient development of the hypothalamic–pituitary axis, an immaturity of synthesis and metabolism of thyroid hormones, and an increase in thyroid hormone requirement needs for thermogenesis and diseases of preterm infants [42,43]. Furthermore, Gu et al. [44] found a higher frequency of premature babies among CH patients with DS (33.7%) compared to both that detected in CH patients without DS (12.2%) and that recorded in the nationwide live birth (28.7%), suggesting that the DS condition, which is a well-established risk factor of prematurity [45], accounts for the significantly increased frequency of CH in preterm infants.

No statistical difference between the proportion of males and females was observed in DS children with CH and OH in our cohort, in agreement with previous studies [8,31]. This equal distribution by sex is also consistent with what we found in the sample of liveborn infants without congenital anomalies. Conversely, previous studies reported a female preponderance for both CH (female-to-male CH ratio = 2.1) and OH (female-to-male OH ratio = 8–9) in the general population [16,17]. 

In this study, we also explored the association between hypothyroidism and CHDs, showing that DS children with CH had a higher, although not statistically significant, risk of developing CHDs. This result is consistent with the hospital-based study by Calcaterra et al. who reported a significant association between CH and the occurrence of any congenital anomalies in patients with DS [8]. 

An increased risk of OH was also observed in DS children with VSD, and this finding can be explained considering that VSD is the most frequent congenital cardiac anomaly in children including those with DS and that hemodynamically unstable VSD can easily be associated with thyroid dysfunction, even SH [35,46]. 

The increased risk of CHDs in children with DS and CH may be explained considering that the development of the embryonic thyroid is closely associated with the development of the heart [21]. In iodine-sufficient countries, most cases (about 80%) of CH are caused by a failure during gland organogenesis, with thyroid hypoplasia described as a main characteristic of DS [16,18,40,47]. However, although thyroid dysgenesis is usually sporadic, in a minority of subjects (2–5%), a mutation in genes involved in thyroid gland formation may occur [11,48]. Some transcriptional factors (e.g., NKX2.5, JAG1) play a crucial role in the organogenesis of the heart and great vessels, in addition to thyroid and parathyroid glands [11,48]. In humans, NKX2.5, expressed in myocardium and pharyngeal endoderm at early stages of embryogenesis, is a key regulator of normal heart morphogenesis, myogenesis, and function mutations in NKX2–5 have been documented in patients with various subtypes of CHDs [49,50]. Heterozygous mutations in NKX2.5 have also been associated with ectopy or athyreosis of the thyroid gland in humans, though with conflicting results [51,52,53,54]. Regarding JAG1, variations in this gene have been related to the pathogenesis of various thyroid disorders, including CH [55,56]. Beyond genetic factors, teratogen exposure to multiple organs, consanguinity, or iodine deficiency during organogenesis may result in an increased incidence of extrathyroidal anomalies associated with CH [25]. The tight association between CH and CHDs may result in the higher estimate of CH prevalence (1%) we found among children with CHDs (with or without DS; data not shown) compared to that observed in control subjects without congenital anomalies (0.07%), which confirms the previously reported prevalence ratio of 10:1 for the occurrence of CH between patients with CHDs and the general population [57]. Additionally, the increased prevalence (between 8.4 and 59%) of major congenital anomalies, in particular CHDs, in CH infants compared to that in the general population [21,23,25,58] suggests an early impairment in embryonal development with the involvement of different organs [23].

The results presented in this study also appear to reinforce the hypothesis of a strict relationship between DS and CH in increasing the risk for CHDs, with chromosomal abnormalities, in particular DS, possibly representing an important etiological factor for congenital anomalies in CH patients. This assumption was suggested by Gu et al. [44] who, for the first time, demonstrated DS-related differences in the incidence of extrathyroidal malformations, in particular CHDs, in subjects with CH in a retrospective hospital-based study. Furthermore, due to thyroid dysregulation which would be directly related to the trisomy of chromosome 21, even a mild increase in TSH levels at birth could lead to a higher frequency of CHDs in DS patients [59,60]. To try to elucidate the close association between DS and CH, we should consider that the extra copy of chromosome 21 (HSA21), or part of it, is associated with a variety of manifestations, including pathologic conditions [61]. In particular, the “Down syndrome critical region (DSCR)”, considered as one of the main candidate regions to be responsible for most DS features, contains important candidate genes such as the dual-specificity tyrosine (Y)-phosphorylation regulated kinase 1 A (DYRK1A), the overexpression of which is believed to affect early thyroid morphogenesis [62,63]. Along with the overexpression of DYRK1A, which could also reduce the availability of tyrosine, decreased levels of selenium, an element not only implicated in thyroid hormone synthesis and metabolism but—as cofactor of glutathione peroxidase and thioredoxin reductase—also in the protection of biosynthetic processes by oxygen free radical toxicity have been reported in DS [61,64]. Furthermore, an excess of Cu/Zn superoxide dismutase activity, another enzyme coded in DSCR, has been suggested to contribute to the chronic oxidative stress observed in individuals with DS [61,65]. Overall, these factors might be related to the hypofunction of the thyroid gland, indicated by the lowered free T4 and increased TSH measured in these patients [61,64].

In summary, if this study provides insights into the epidemiology of hypothyroidism in DS patients, future multicenter studies involving a larger sample size of patients with DS could produce more accurate prevalence estimates useful for the timely management of patients with thyroid disorders. Meanwhile, information on genetic–environmental networks (i.e., maternal and neonatal exposures and newborn genetic mutations), expanding knowledge on the etiology of CH and the underlying mechanisms implicated in the increased frequency of congenital anomalies, particularly CHDs, in DS patients with CH may be relevant to prevent some types of thyroid dysgenesis by modulating these cofactors. Also, the development of “hybrid” approaches, integrating information from a population-based registry and hospital discharge records with data from medical records, may allow more accurate diagnoses on congenital (both permanent and transient) and overt hypothyroidism. At the same time, the results of thyroid function tests would play a key role in evaluating the association between SH and CHDs to verify whether, like CH, this condition increases the risk of CHDs in patients with DS compared to those without SH. 

### Strength and Limitations

The major strength of this study is the design based on population-based registries, which may represent a useful tool for exploring the epidemiology of hypothyroidism in patients with DS and associated anomalies, thus playing a fundamental role in public health surveillance. Indeed, compared to hospital-based registries, essential for clinical research, patient care and management, population-based registries include all patients residing in a defined geographical area and are the only tool that enables the production of population-based epidemiological indicators (e.g., prevalence). The current research monitored a resident population of approximately 3.7 million inhabitants over a 14-year period, long enough to provide sufficiently precise estimates of prevalence. In addition, to the best of our knowledge, this is the first population-based study to evaluate the association between clinical hypothyroidism (both congenital and acquired) in DS and the occurrence of CHDs in these patients. 

This study also has some limitations. First, DS children with OH were selected based on pharmaceutical prescriptions (i.e., treatment with L-T4), but it cannot be excluded that DS patients with SH may also have been treated with L-T4. In fact, there is still debate as to whether patients with SH should be treated, with most investigators pointing out there are no benefits of L-T4 on growth and motor or mental development [36]. Second, as for the identification of cases with transient CH, we could not verify whether these subjects had normal thyroid function tests one month or more after the discontinuation of L-T4 and off therapy. 

## 5. Conclusions

In this population-based study, we estimated a prevalence of 11.4% and 12.7% for CH and OH, respectively, which confirmed the much higher frequency of these conditions in DS compared to the general population, without significant differences by sex. We also found that children with DS and CH have a higher risk of CHDs than DS patients without CH, while VSDs in DS children are associated with OH. This study provides insights into the epidemiology of hypothyroidism in DS patients and associated anomalies, representing a first step towards future multicenter studies able to integrate information from population-based registries with clinical data to improve the accuracy of prevalence estimates and explore further thyroid disorders in DS.

## Figures and Tables

**Table 1:** Comparison by period and sex in the prevalence of congenital hypothyroidism.

Total				Males				Females	
Period	Number of Cases	Prevalence (95% CI)	*p*	Number of Cases	Prevalence (95% CI)	*p*	Number of Cases	Prevalence (95% CI)	*p*
2003–2009	8	7.4% (3.2–14.6%)	**<0.0001**	4	6.3% (1.7–16.2%)	**0.0001**	4	8.9% (2.4–22.7%)	**0.0024**
2010–2017	18	15.0% (8.9–23.7%)		7	13.5% (5.4–27.7%)		11	16.2% (8.1–28.9%)	
2003–2017	26	11.4% (7.4–16.7%)		11	9.6% (4.8–17.1%)		15	13.3% (7.4–21.9%)	

*p*-value < 0.05 set as statistically significant is in bold.

**Table 2:** Comparison by period and sex in the prevalence of overt hypothyroidism.

	Total			Males				Females	
Period	Number of Cases	Prevalence (95% CI)	*p*	Number of Cases	Prevalence (95% CI)	*p*	Number of Cases	Prevalence (95% CI)	*p*
2003–2009	15	13.9% (7.8–22.9%)	0.188	5	8.0% (2.6–18.5%)	**0.045**	10	22.2% (10.6–40.9%)	**0.0008**
2010–2017	14	11.7% (6.4–19.6%)		86	11.5% (4.2–25.1%)		8	11.8% (5.1–23.2%)	
2003–2017	29	12.7% (8.5–18.3%)		11	9.6% (4.8–17.1%)		18	15.9% (9.4–25.2%)	

*p*-value < 0.05 set as statistically significant is in bold.

**Table 3:** Main features of Down syndrome patients with congenital and overt hypothyroidism.

Variable Number (%)	DS Children without CH and OH §	Congenital Hypothyroidism		Overt Hypothyroidism	
	n = 165	n = 26	*p* *	n = 29	*p* *
Sex					
Males	86 (52.1)	11 (42.3)	0.352	11 (37.9)	0.159
Preterm infants **	26 (18.8)	11 (42.3)	**0.009**	5 (18.5)	0.969
Maternal age					
<35	58 (38.4)	7 (26.9)	0.516	7 (25.0)	0.364
≥35–<40	50 (33.1)	10 (38.5)		10 (35.7)	
≥40	43 (28.5)	9 (34.6)		11 (32.3)	
Paternal age					
<35	39 (30.5)	3 (15.8)	0.199	6 (25.0)	0.661
≥35–<40	31 (24.2)	3 (15.8)		8 (33.3)	
≥40	58 (45.3)	13 (68.4)		10 (41.7)	
Maternal BMI					
<19	12 (9.1)	1 (3.8)	0.716	2 (7.4)	0.982
≥19–<25	76 (57.5)	18 (69.2)		17 (63.0)	
≥25–≤30	34 (25.8)	5(19.2)		7 (25.9)	
>30	10 (7.6)	2 (7.7)		1 (3.7)	
Maternal nationality					
Italy and Developed Countries	112 (75.2)	22 (84.6)	0.294	24 (88.8)	0.117
Maternal education					
Elementary and lower secondary	41 (30.4)	4 (15.4)	**0.042**	5 (19.2)	0.429
Upper secondary	56 (41.5)	8 (30.8)		14 (53.8)	
Tertiary	38 (28.1)	14 (53.8)		7 (26.9)	
Maternal smoking during pregnancy					
Smokers	13 (10.7)	1 (4.8)	0.396	4 (16.7)	0.410
Associated CHDs					
Yes	52 (31.5)	15 (57.7)	**0.009**	14 (48.3)	0.079

§ Cases with transient congenital hypothyroidism were excluded (N = 8). *p*-value < 0.05 set as statistically significant is in bold. * *p*-value in the comparison with children with Down syndrome without both congenital and overt hypothyroidism. ** Preterm infants: gestational age < 37 weeks. Abbreviations: BMI: body mass index; CHDs: congenital heart defects; DS: Down syndrome; CH: congenital hypothyroidism, OH: overt hypothyroidism.

**Table 4:** Association between congenital and overt hypothyroidism with congenital heart defects in patients with Down syndrome.

DS Children n = 228		Congenital Hypothyroidism n = 26					Overt Hypothyroidism n = 29				
Anomalies	Number (%)	Number (%)	OR (95%CI)	*p*	adjOR (95%CI) *	*p*	Number (%)	OR (95%CI)	*p*	adjOR (95%CI) *	*p*
Total CHDs	84 (36.8%)	15 (57.7%)	**2.96 (1.27–6.89)**	**0.012**	2.24 (0.90–5.58)	0.082	14 (48.2%)	2.03 (0.91–4.51)	0.085	1.68 (0.55–2.98)	0.254
Severe CHDs	27 (11.8%)	5 (19.2%)	1.94 (0.65–5.79)	0.232	1.47 (0.45–4.76)	0.518	3 (10.3%)	0.94 (0.26–3.43)	0.928	0.60 (0.12–2.92)	0.525
VSD	37 (16.2%)	3 (11.5%)	0.85 (0.23–3.06)	0.801	0.55 (0.14–2.18)	0.399	10 (34.5%)	**3.42 (1.41–8.31)**	**0.009**	**3.07 (1.15–8.15)**	**0.025**
AVSD	20 (8.7%)	4 (15.4%)	2.12 (0.64–7.10)	0.221	1.45 (0.40–5.25)	0.568	2 (6.9%)	0.87 (0.18–4.05)	0.853	0.78 (0.16–3.86)	0.760
ASD	19 (8.3%)	2 (7.7%)	0.90 (0.19–4.20)	0.892	0.77 (0.14–4.27)	0.767	3 (10.3%)	1.24 (0.33–4.63)	0.749	0.81 (0.16–4.13)	0.806
PDA	15 (6.6%)	4 (15.4%)	2.82 (0.81–9.76)	0.102	2.60 (0.65–10.32)	0.175	1 (3.4%)	0.55 (0.07–4.50)	0.551	0.57 (0.07–4.86)	0.605

*p*-value < 0.05 set as statistically significant is in bold. * Model adjusted for sex, gestational age and maternal education. Abbreviations: adjOR: adjusted odds ratio; ASD: atrial septal defect; AVSD: atrioventricular septal defect; CHDs: congenital heart defect; DS: Down syndrome; PDA: patent ductus arteriosus in full term infants; VSD: ventricular septal defect.

## Data Availability

The data supporting the findings of this study are available from Regione Toscana but restrictions apply to the availability of these data, which were used under license for the current study, and therefore are not publicly available. Data are, however, available from the authors upon reasonable request and with the permission of Regione Toscana. Requests to access the datasets should be directed to Regione Toscana, https://www.regione.toscana.it/ (accessed on 12 March 2024).

## Abbreviations

ASD	Atrial septal defect
BMI	Body mass index
CH	Congenital hypothyroidism
CHD	Congenital heart defect
DS	Down syndrome
HDD	Hospital discharge database
OH	Overt hypothyroidism
RCDT	Registry of Congenital Defects of Tuscany
RRDT	Registry of Rare Diseases of Tuscany
SH	Subclinical hypothyroidism
T4	Thyroxine
TSH	Thyroid-stimulating hormone
VSD	Ventricular septal defect
